# Comment on: Transrectal prostate biopsy complications: a prospective single center study in a mid-income country

**DOI:** 10.31744/einstein_journal/2026CE2121

**Published:** 2026-02-12

**Authors:** Hüsnü Tokgöz, Özlem Tokgöz

**Affiliations:** 1 Private Academy Hospital Departments of Urology Mersin Turkey Departments of Urology, Private Academy Hospital, Mersin, Turkey.; 2 Radiology Mersin Turkey Radiology, Mersin, Turkey.

Dear Editor,

We read the article by Dr. Schollemberg et al. with great interest.^(1)^ In their prospective cohort study, they assessed the complications and associated risk factors following transrectal ultrasound-guided prostate biopsy (TRUSB) at a Brazilian public reference center. A total of 1,043 consecutive patients were assessed using the Global Prevalence of Infections in Urology form, and complications were classified based on the Clavien-Dindo system. Most bleeding events were mild (Grade 1), whereas 1.5% were of Grades 2–3 and were significantly associated with hypertension, younger age, and anticoagulant use (p<0.001). Infectious complications occurred in 4.7% of patients (Grades 2, 3, and 4: 0.5, 3.6, and 0.6%, respectively), with indwelling catheter use, recent urinary tract infection (p<0.001), and quinolone use (odds ratio [OR] = 3.01, 95% confidence interval (95%CI) 1.15–7.80, p=0.03) identified as risk factors. Urinary retention was observed in 4.1% of cases and was associated with severe lower urinary tract symptoms (p=0.009), prostate volume >89mL (p=0.001), and prostatic protrusion ≥10mm (p=0.001). This study demonstrates that although life-threatening complications are uncommon, TRUSB should not be considered completely risk-free.

Recently, we assessed oxidative stress alterations—specifically thiol/disulphide homeostasis—in men undergoing TRUSB.^(2)^ Twenty-two patients with abnormal digital rectal examination and/or prostate-specific antigen (PSA) levels >4ng/mL underwent 12-core TRUSB. Serum samples collected before and after the procedure were analyzed for thiol/disulfide parameters. The results demonstrated significant post-biopsy reductions in serum native and total thiol levels, whereas disulfide concentrations and associated ratios remained unaltered. No associations were observed between the oxidative markers and PSA levels, prostate volume, or histopathology. However, patient age was significantly associated with native and total thiol levels, indicating that older age at biopsy may increase the risk of procedure-induced morbidity and oxidative stress. In contrast, Schollemberg et al. reported a significant association between younger age and a higher risk of hemorrhagic complications, possibly because of better vascularity in younger patients and/or biopsy strategies involving larger tissue sampling. Additionally, infectious complications were observed in approximately 5% of patients, with significant associations identified for recent quinolone use. Similarly, Wagenlehner et al., in their Global Prevalence of Infections in Urology study, highlighted quinolone use as a contributing factor to increasing rates of urosepsis in patients undergoing prostate biopsy.^(3)^ Additionally, we conducted a study assessing the effect of long-term quinolone administration before TRUSB on post-procedure sepsis.^(4)^ Among 558 patients, 205 received levofloxacin (500mg daily) for three weeks before biopsy, whereas 353 received no antibiotic treatment. Sepsis occurred in 17 patients (3.0%), with a significantly higher rate in the quinolone group than that in the non-antibiotic group (5.4 *versus* 1.7%; p=0.0297; OR = 3.28, 95% CI: 1.10–10.13). Temporal analysis indicated an increasing trend in sepsis during the latter study period, although this was not statistically significant. *Escherichia coli* was the predominant pathogen, universally resistant to fluoroquinolones—55.6% produced extended-spectrum β-lactamases, and one patient had methicillin-resistant *Staphylococcus epidermidis*. Although Schollemberg et al. did not report the bacterial types isolated from the urine cultures of the 27 patients (4.7%) who developed infectious complications, including four patients (0.6%) with urosepsis, their data also indicate that prolonged quinolone use before TRUSB may increase the risk of resistant microorganism-caused post-biopsy sepsis, emphasizing the significance of judicious antibiotic prophylaxis and antimicrobial stewardship in urological practice.

In conclusion, TRUSB remains a valuable method for diagnosing prostate cancer. Currently, fusion biopsies that associate prostatic magnetic resonance imaging findings with transrectal or transperineal approaches enable more precise sampling of targeted regions while reducing unnecessary biopsies. Additionally, assessing oxidative stress in patients undergoing fusion biopsy may provide further insights into the biological effects of the procedure. The use of prophylactic quinolones before biopsy—as observed in both our study and the study by Schollemberg et al.—may increase the risk of resistant microorganism-caused post-biopsy infections. Age-matched prospective studies are required to confirm these effects because patient age can directly affect the incidence and severity of post-biopsy complications. Rising antibiotic resistance is likely to prompt urologists and infectious disease specialists to reassess prophylactic protocols before interventional procedures, such as TRUSB, potentially resulting in updates to clinical guidelines.

## Data Availability

The underlying content is contained within the manuscript.
